# Dosimetric impact and detectability of multi‐leaf collimator positioning errors on Varian Halcyon

**DOI:** 10.1002/acm2.12677

**Published:** 2019-07-11

**Authors:** Skylar S. Gay, Tucker J. Netherton, Carlos E. Cardenas, Rachel B. Ger, Peter A. Balter, Lei Dong, Dimitris Mihailidis, Laurence E. Court

**Affiliations:** ^1^ Department of Radiation Physics The University of Texas MD Anderson Cancer Center Houston TX USA; ^2^ The University of Texas MD Anderson Cancer Center UTHealth Graduate School of Biomedical Sciences Houston TX USA; ^3^ Radiation Oncology, Hospital University of Pennsylvania Philadelphia PA USA; ^4^ Department of Imaging Physics The University of Texas MD Anderson Cancer Center Houston TX USA

**Keywords:** dual‐layer, Halcyon, head and neck, MLC, quality assurance, VMAT

## Abstract

The purpose of this study is to investigate the dosimetric impact of multi‐leaf collimator (MLC) positioning errors on a Varian Halcyon for both random and systematic errors, and to evaluate the effectiveness of portal dosimetry quality assurance in catching clinically significant changes caused by these errors. Both random and systematic errors were purposely added to 11 physician‐approved head and neck volumetric modulated arc therapy (VMAT) treatment plans, yielding a total of 99 unique plans. Plans were then delivered on a preclinical Varian Halcyon linear accelerator and the fluence was captured by an opposed portal dosimeter. When comparing dose–volume histogram (DVH) values of plans with introduced MLC errors to known good plans, clinically significant changes to target structures quickly emerged for plans with systematic errors, while random errors caused less change. For both error types, the magnitude of clinically significant changes increased as error size increased. Portal dosimetry was able to detect all systematic errors, while random errors of ±5 mm or less were unlikely to be detected. Best detection of clinically significant errors, while minimizing false positives, was achieved by following the recommendations of AAPM TG‐218. Furthermore, high‐ to moderate correlation was found between dose DVH metrics for normal tissues surrounding the target and portal dosimetry pass rates. Therefore, it may be concluded that portal dosimetry on the Halcyon is robust enough to detect errors in MLC positioning before they introduce clinically significant changes to VMAT treatment plans.

## 1 INTRODUCTION

Modern radiation therapy uses the multi‐leaf collimator (MLC) heavily to shape and control the beam delivered to the patient during treatments using approaches such as intensity‐modulated radiation therapy (IMRT) and volumetric modulated arc therapy (VMAT). Because the precision of leaf positioning directly impacts both the effectiveness of the radiation therapy and the patient’s well‐being, it is critical that errors because of misaligned or misconfigured MLC equipment, or errors arising from underperforming MLC components such as motors not receiving the correct voltage, be avoided whenever possible and the effects of these errors known. The dosimetric impact of these positioning errors has been extensively examined in the literature.^1^, ^2^, ^3^, ^4^, ^5^ Furthermore, possible quality assurance (QA) errors that are inherent to MLC design have been studied by American Association of Physicists in Medicine Task Groups (TG) 50 and 142, which have recommended best practices to avoid or minimize the consequences of these errors.^6^, ^7^

In particular, some studies have compared dosimetric differences in plans with random MLC leaf errors (where leaves are shifted some random amount within provided parameters) with differences in plans with systematic MLC leaf errors (where leaves are shifted identical amounts). Although both error classes have been shown to unfavorably impact treatment plans, systematic errors are reported to be more significant than random errors of the same magnitude.^1^, ^2^

However, all such studies to date have used a linear accelerator with a single‐layer MLC design, commonly for which upper or lower collimation jaws are supplemented or replaced by the MLC, although various MLC designs exist.^6^, ^8^ These variations in MLC design and geometry introduce potentials for uncertainty which may be further compounded by such variables as leaf size and design, the introduction or removal of a field penumbra, or restricting treatment modalities.^2^ The recently introduced Halcyon linear accelerator instead uses a jaw‐free, dual‐layer MLC design (Varian Medical Systems, Palo Alto, CA, USA). Thus, existing knowledge may not be directly translatable to the unique conditions that the Halcyon provides. In particular, the dual‐layer MLC design greatly reduces inter‐leaf dose leakage and low dose spillage that may have contributed to the overall dosimetric changes reported by previous studies.^9^, ^10^, ^11^

The purpose of the current study was to evaluate the dosimetric impact of both random and systematic errors in the leaf positions of Halcyon’s dual‐layer MLC, to examine the correlation between clinically significant dosimetric changes and QA pass rates, and to estimate our ability to consistently detect these errors with portal dosimetry before the errors become clinically significant. This is the first study of its kind to examine such an impact from dual‐layer MLC errors.

## 2 MATERIALS AND METHODS

### 2.1 Linear accelerator

**Figure 1:**
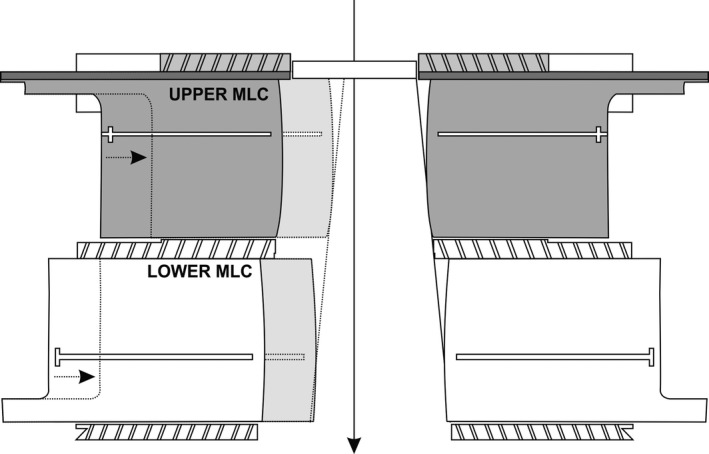
Halcyon multi‐leaf collimation system cross section. Beam direction is indicated by the arrow. Diagram courtesy of Varian Medical Systems.

A preclinical version of the Halcyon was used for the current study. The Halcyon does not use moving jaws for beam collimation; instead, initial collimation is performed by fixed primary and secondary collimators and the beam is further shaped by a novel two‐layered MLC system (Fig. 1). MLC leaves on the Halcyon, like other Varian linear accelerators, move along a straight line and have rounded ends. The MLC layer proximal to the source contains 56 leaves of 1 cm width distributed across the X1 and X2 banks, or 28 leaves per bank, and the layer distal to the source uses 58 leaves, or 29 leaves per bank. The distal layer leaves are staggered by 0.5 cm relative to the proximal leaves to reduce interleaf dose leakage. The Halcyon provides MLC speeds of up to 5.0 cm per second and dose rates of 800 monitor units per minute.^12^ Although kilovoltage imaging is now available in some markets, the unit in the current study used only a 6‐MV flattening filter‐free beam for both imaging and treatment.

### 2.2 Patient data

The current study used 11 physician‐approved head and neck VMAT plans for treatments delivered at Penn Medicine Center at the University of Pennsylvania. Four of these plans contained two treatment arcs, four contained three arcs, and the other three contained four arcs. The median dose per fraction was 2 Gy (range 1.8–2.12 Gy), and the median number of treatment fractions was 30 (range 14–35). All treatment plans for Halcyon include one or more imaging fields.^12^ These were unchanged and did not contribute significantly to the current study. All data were obtained with the appropriate institutional review board approvals and data transfer agreements.

All patient plans contained the following physician‐contoured clinical structures: brainstem, eyes, lenses, right parotid, spinal cord and expanded spinal cord, and high‐risk planning target volume (PTV) structures, of which all but eyes and lenses were selected (Table 1). When applicable, low‐ and mid‐risk PTVs were also selected. Other structures included optic chiasm, optic nerves, left parotid, and submandibular glands. Because these structures were not consistently contoured for all patients, they were not directly considered in the current study.

**Table 1:** Dose***–***volume histogram metrics evaluated for all normal structures.

Structure	Dose metrics	Volume metrics 1	Volume metrics 2
Brainstem	D_max_	V_54 Gy_	
Spinal cord	D_max_	V_45 Gy_	
Expanded spinal cord	D_max_	V_45 Gy_	
Right parotid	D_mean_	V_30 Gy_	
High‐risk PTV^a^		V_98%_	V_95%_
Mid‐risk PTV		V_98%_	V_95%_
Low‐risk PTV		V_98%_	V_95%_

D_max_ is defined as the maximum point dose to the tissue, D_mean_ is the mean point dose to the tissue, V_54 Gy_ is the volume of tissue receiving 54 Gy, V_45 Gy_ is the volume of tissue receiving 45 Gy, V_30 Gy_ is the volume of tissue receiving 30 Gy, V_98%_ is the volume of tissue receiving 98% of the prescription dose, and V_95%_ is the volume of tissue receiving 95% of the prescription dose.

a Planning target volume.

### 2.3 Error simulation

In‐house software was used to introduce various controlled errors within each treatment plan. Plans were exported in DICOM format from the treatment planning system to a local directory. Pydicom was then used to read and edit leaf position sequences directly and create a new treatment plan associated with the corresponding original, unmodified treatment plan.^13^

Four magnitudes of random error were simulated by adding an array containing a random distribution uniformly sampled within ±3, ±5, ±7, or ±10 mm to each control point. These values were selected based on our own preliminary work using the same Halcyon device which showed that smaller errors did not have noticeable impact. This is supported by the work of previous authors on single‐layer MLCS which showed there is no significant change in dose–volume histogram (DVH) for random errors up to 2 mm.^2^ To avoid potential biases or unintentional duplication, a new random distribution was generated for each treatment arc and each plan, so that identical random distributions were never added to any two arcs or plans. In contrast, identical symmetric shifts of 3, 5, 7, or 10 mm were added to MLC leaf positions to simulate four degrees of systematic errors, where all leaves were shifted equal amounts in the same direction relative to the beam isocenter. Repeating both processes for all 11 original plans provided a total of 99 plan variants: 11 unmodified, 44 with random errors, and 44 with systematic errors. After errors were added, appropriate adjustments were made to avoid overlapping leaves, dynamic leaf gaps, or leaves being shifted outside the collimator boundaries. When errors were added to treatment plans, average MLC leaf shifts, and the standard deviation of each iteration were automatically recorded by the in‐house software, and these records were later reviewed and validated to ensure that they were within expected values.

Once all 99 plan variants were generated, they were imported into a research version of Eclipse and the dose distribution was recalculated for all plans. A QA verification plan was then generated for each treatment plan and later delivered on the Halcyon through the Treatment Mode workspace and captured using the Halcyon’s opposing portal dosimetry device. Initially, each verification plan was delivered twice, and individual fractions were compared for variations, but after analysis showed no noticeable differences between fractions, the remaining plans were delivered once.

### 2.4 Portal image detection

The Portal Dosimetry workspace (Varian Medical Systems) was used to perform gamma analysis as described in the literature.^14^ For the current study, low‐dose threshold was set to 10% and no region‐of‐interest was set (vendor default settings). For each of the 11 patients, the predicted fluence of that patient’s unmodified plan was taken as the reference to accurately simulate the effect of MLC positioning errors during beam delivery.

Gamma evaluation was performed with a combination of two absolute dose difference criteria, 3% and 2%, as well as three distance‐to‐agreement criteria, 3, 2, and 1 mm, for a total of four different indices: 3%/3 mm (in common use), 3%/2 mm (proposed by American Association of Physicists in Medicine Task Group 218 [TG‐218]), 2%/2, and 2%/1 mm.^15^ Because all plans contained at least two treatment arcs, each plan’s overall agreement was taken as the mean of the agreement values for each arc, where perfect agreement was 100% and complete disagreement was 0%, within the provided evaluation criteria.

To determine whether a given plan had passed or failed portal dosimetry QA, two different criteria were examined:

1. If the overall percent agreement was 95% or higher, the plan passed; otherwise, it failed.
2. If the overall percent agreement of any of the modified plans was no lower than 2% below the lowest mean percent agreement for the unmodified plans, the plan passed. For example, if the lowest pixel agreement of any of the 11 unmodified treatment plans was 93% for a 3%/2 mm evaluation, any plan with 91% or higher overall agreement passed. This approach was used to more closely examine the effect of adding MLC errors on the QA results beyond the unmodified (“error‐free”) plan.

### 2.5 Dose–volume histogram metric evaluation and normalization

To evaluate the impact of the MLC positioning errors on the dose delivered to the patient, DVH metrics for all normal structures were calculated using Eclipse for all 99 treatment plans, and text files containing these distributions were exported. In‐house software was then used to extract target dose and volume coverage metrics for the normal tissue structures. Additionally, values for the volume of tissue receiving 98% and 95% of the prescription dose (V_98%_ and V_95%_ values) were extracted for high‐, mid‐, and low‐risk PTVs. The dose to normal tissues for each patient was normalized to the dose delivered to that patient’s unmodified treatment plan. The volume coverage of normal tissues for each patient was measured in cubic centimeters, and volume coverage for PTVs was measured as a non‐normalized percentage. Notable DVH metrics for the primary normal structures we considered are shown in Table 1, and clinically significant errors were defined as >5% change to these structures.

### 2.6 DVH metric correlation to portal dosimetry pass rate

Pearson’s correlation coefficients (r‐values) and *P*‐values were used to determine the correlation between clinically significant changes to normal structures nearby or surrounding the target and the portal dosimetry pass rate of all plans at all gamma indices. High‐ and moderate correlation were defined to be |r|≥ 0.75 and |r| ≥ 0.4, respectively, and statistically significant *P*‐values defined to be less than 0.03, based on similar values in the literature.^16^

## 3 RESULTS

### 3.1 Changes to DVH metrics

**Figure 2:**
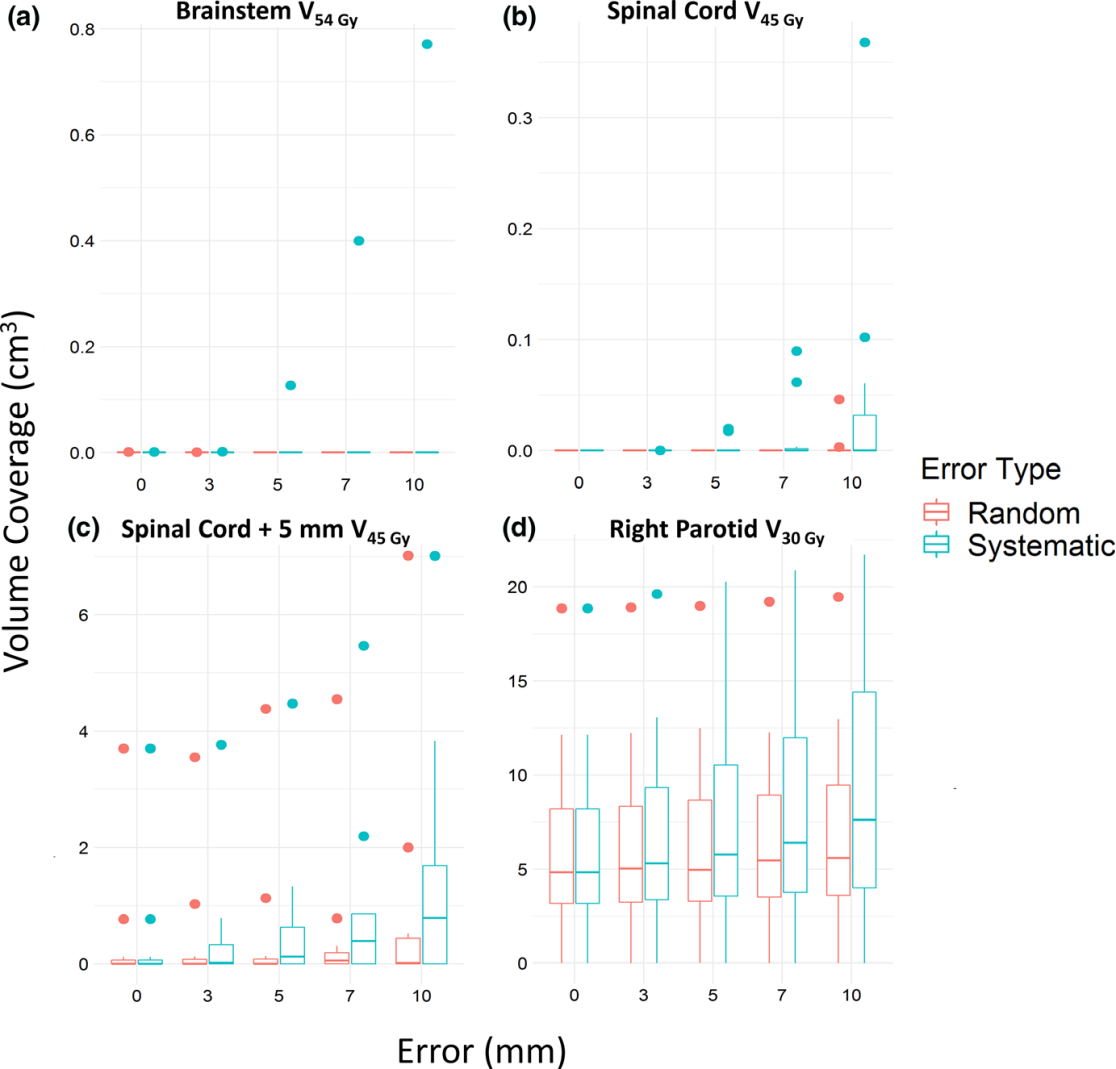
Change in normal structure volume covered. The volume of normal structures covered increased as errors increased, especially for systematic errors. (a) Brainstem volume receiving ≤54 Gy. (b) Spinal cord volume receiving ≤45 Gy. (c) Expanded spinal cord volume receiving ≤45 Gy. (d) Right parotid volume receiving ≤30 Gy. Points indicate outliers

**Figure 3:**
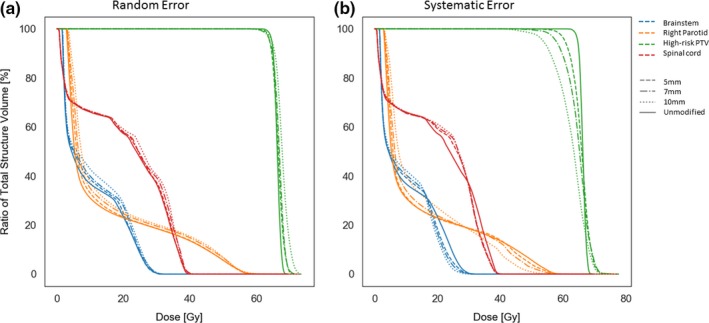
Comparison of cumulative dose***–***volume histogram at (a) random and (b) systematic errors. Dose***–***volume histogram shown is for a single patient. The 3 mm error is omitted owing to viewing constraints, but it behaved according to the overall trend. PTV, planning target volume.

**Figure 4:**
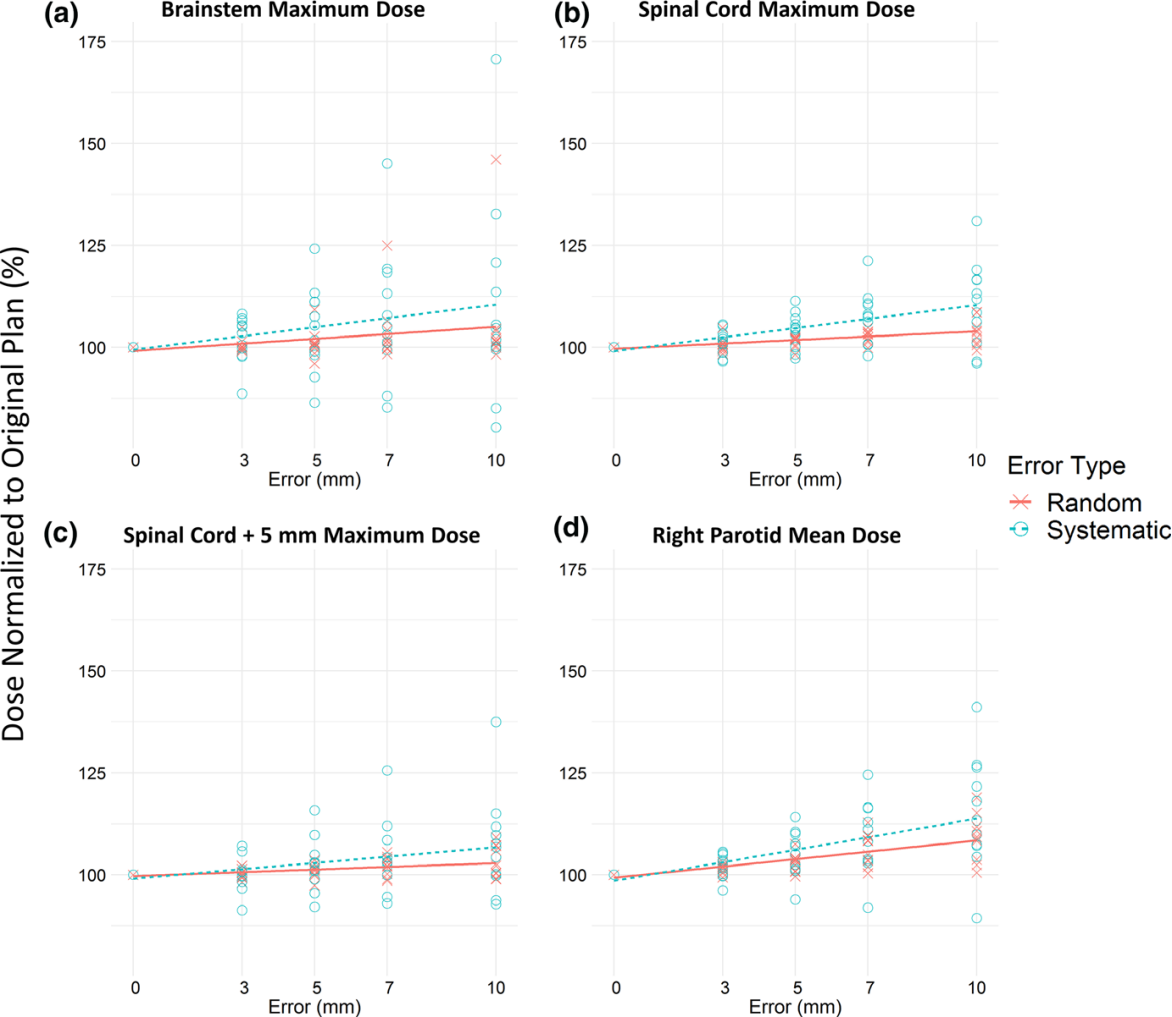
Maximum or mean dose changed as errors increased. Systematic errors changed the dose more significantly than did random errors for (a) maximum dose to the brainstem, (b) maximum dose to the spinal cord, (c) maximum dose to the expanded spinal cord, and (d) mean dose to the right parotid. Dose to each structure was normalized as a percentage of the dose delivered to the structure of the corresponding unmodified plan. Points represent individual plans at the indicated amount of multi‐leaf collimation error. Trend lines provide a guide for visual inspection.

The impacts of systematic and random errors on the tissue doses to the brainstem, spinal cord, spinal cord expanded by 5 mm, and right parotid are shown in Figs. 2, 3, 4. Other tissues showed similar results. Systematic errors resulted in larger changes to normal structure volume coverage than did random errors (Fig. 2). Changes in volume coverage trended upward as error magnitude increased for both random and systematic errors.

Similarly, systematic errors had a greater effect than random errors on the maximum dose to the brainstem, spinal cord, and expanded spinal cord, as well as on mean dose to the right parotid, as seen in Figs. 3 and 4. The general trend for random and systematic errors was to increase delivered dose, although for some patients, errors resulted in a decrease in dose.

**Figure 5:**
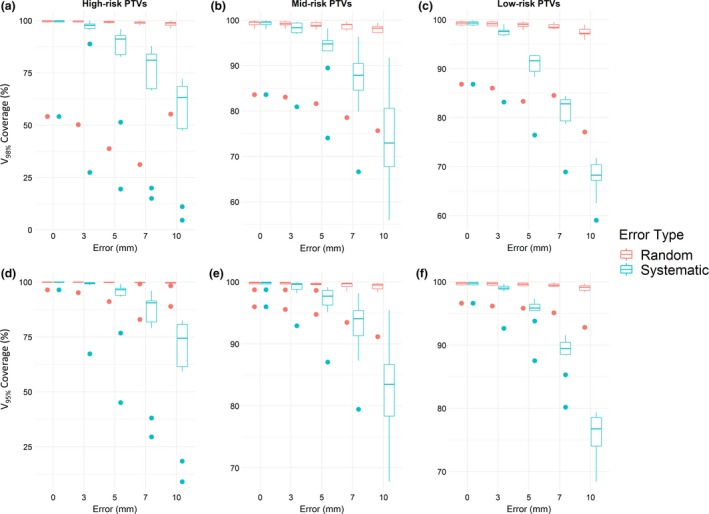
Changes in planning target volume (PTV) coverage as the magnitude of error increased. (a–c) V_98%_ coverage for high‐, mid‐, and low‐risk PTV, respectively. (d–f) V_95%_ coverage for high‐, mid‐, and low‐risk PTV. Points indicate outliers.

Systematic errors also had a greater effect than random errors on PTV volume coverage evaluated at 95% and 98% of the prescription dose (Fig. 5). High‐risk PTV was more substantially impacted than mid‐ and low‐risk PTVs. Mean volume coverage trended downward as the magnitude of errors increased for both random and systematic errors.

### 3.2 Clinical impact

**Figure 6:**
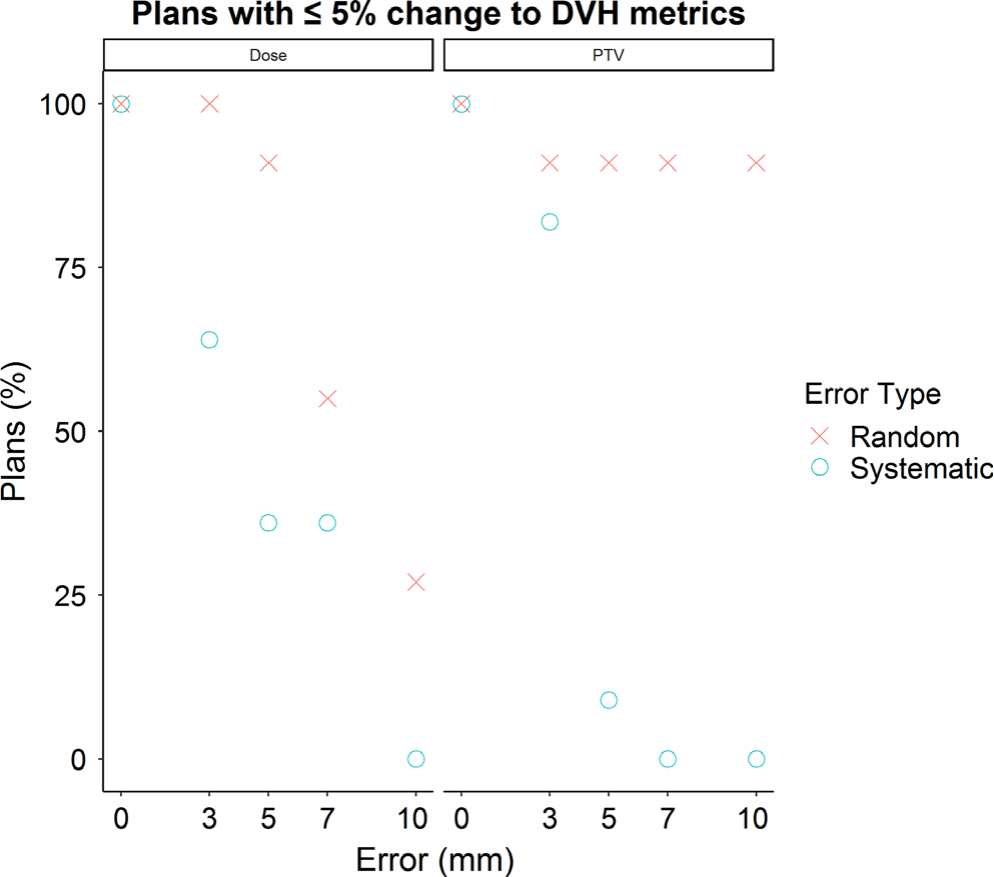
Percentage of error plans with <5% change to dose***–***volume histogram metrics for structures listed in Table 1. PTV, planning target volume.

Systematic errors rapidly introduced clinically significant changes to the DVH metrics listed in Table 1. Using pass/fail criteria where plans with >5% change to DVH metrics (“clinically significant errors”) fail evaluation, most plans with 5 mm or higher systematic error failed (Fig. 6). Conversely, although random errors also caused some degree of clinically significant errors, the effect was less pronounced. Most plans with random errors did not exhibit a clinically significant change to PTV covered, with the pass rate plateauing at 91% for errors between 3 and 10 mm. The pass rate for dose metrics decreased fairly linearly for plans with random errors; all 3 mm error plans and 27% of 10 mm error plans passed.

### 3.3 Portal dosimetry pass rates, and error detection

**Figure 7:**
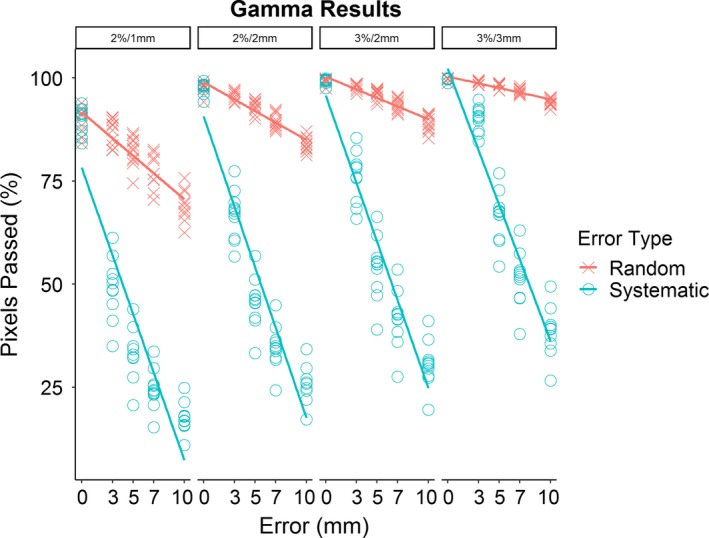
Mean gamma score for agreement between predicted dose distribution for unmodified plans and delivered dose distribution (for both unmodified and error plans) at four indicated gamma indices.

**Figure 8:**
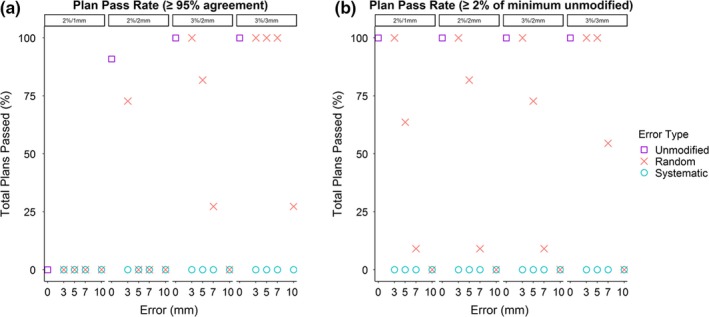
Portal dosimetry pass rates for plans at indicated gamma indices, using the two pass/fail criteria described in Methods. Points represent mean pass rate for all plans at the indicated multi‐leaf collimation error. Regardless of pass/fail criteria, systematic errors rapidly cause plans to fail gamma evaluation, while failure for plans with random errors depends more on gamma indices and evaluation criteria. (a) Overall pass rate for the first pass/fail criteria (e.g., ≥95% mean pixel agreement with predicted dose to pass). (b) Pass rate for the same plans when using the secondary pass/fail criteria (e.g., mean pixel agreement no lower than 2% of the minimum mean pixel agreement of the unmodified plans to pass).

Unlike the pass rates for DVH metrics, all plans with systematic errors immediately failed portal dosimetry for both classes of pass/fail criteria using any of the specified gamma indices [Figs. 7, 8(a), and 8(b)]. The pass rate for plans with random errors tended to decrease as the magnitude of random errors increased for both classes of pass/fail criteria when evaluated with any of the specified gamma indices. Portal dosimetry pass rates for all four indices are shown in Table 2.

**Table 2:** Observed portal dosimetry pass rates for all four indices.

	3%/3 mm		3%/2 mm		2%/2 mm		2%/1 mm	
	≥95% agreement	2% of unmodified	≥95% agreement	2% of unmodified	≥95% agreement	2% of unmodified	≥95% agreement	2% of unmodified
Unmodified	100.0%	100.0%	100.0%	100.0%	90.9%	100.0%	0.0%	100.0%
3 mm Random	100.0%	100.0%	100.0%	100.0%	72.7%	100.0%	0.0%	100.0%
3 mm Systematic	0.0%	0.0%	0.0%	0.0%	0.0%	0.0%	0.0%	0.0%
5 mm Random	100.0%	100.0%	81.8%	72.7%	0.0%	81.8%	0.0%	63.6%
5 mm Systematic	0.0%	0.0%	0.0%	0.0%	0.0%	0.0%	0.0%	0.0%
7 mm Random	100.0%	54.6%	27.3%	9.1%	0.0%	9.1%	0.0%	9.1%
7 mm Systematic	0.0%	0.0%	0.0%	0.0%	0.0%	0.0%	0.0%	0.0%
10 mm Random	27.3%	0.0%	0.0%	0.0%	0.0%	0.0%	0.0%	0.0%
10 mm Systematic	0.0%	0.0%	0.0%	0.0%	0.0%	0.0%	0.0%	0.0%

The first column for each index is the passing rate where “passing” is defined as ≥95% mean pixel agreement with the predicted dose. The second column for each index is the passing rate where “passing” is defined as mean pixel agreement no lower than 2% of the minimum mean pixel agreement of the unmodified plans.

### 3.4 Correlation between changes to DVH metrics and portal dosimetry pass rates

Using Pearson’s correlation, high (|r| ≥ 0.75) to moderate (|r| ≥ 0.4) correlation in maximum and mean dose DVH metrics and QA pass rates was observed for some structures, particularly those close to the target such as the brainstem, spinal cord, and high‐risk PTV (Table 3). Correlation varied by structure and DVH metric type as well as between random and systematic error. Volume metrics had no meaningful correlation except for those to the PTV in plans with systematic errors, which were moderately correlated with QA pass rates.

**Table 3:** Pearson correlation coefficients (r) and *P* values for the target volume and nearby normal structures.

DVH metric	Gamma indices	Brainstem				Spinal cord				High‐risk PTV			
		Random		Systematic		Random		Systematic		Random		Systematic	
		r	*P*	r	*P*	r	*P*	r	*P*	r	*P*	r	*P*
D_max_	3%/3 mm	−0.34	0.02	−0.27	0.08	−0.59	<0.01	−0.56	<0.01	−0.74	<0.01	−0.57	<0.01
	3%/2 mm	−0.38	0.01	−0.26	0.1	−0.58	<0.01	−0.52	<0.01	−0.75	<0.01	−0.59	<0.01
	2%/2 mm	−0.34	0.03	−0.24	0.12	−0.6	<0.01	−0.52	<0.01	−0.74	<0.01	−0.59	<0.01
	2%/1 mm	−0.38	0.01	−0.23	0.14	−0.53	<0.01	−0.48	<0.01	−0.71	<0.01	−0.58	<0.01
D_mean_	3%/3 mm	−0.84	<0.01	−0.45	<0.01	−0.82	<0.01	−0.54	<0.01	−0.3	0.06	0.66	<0.01
	3%/2 mm	−0.86	<0.01	−0.42	<0.01	−0.85	<0.01	−0.49	<0.01	−0.3	0.05	0.63	<0.01
	2%/2 mm	−0.85	<0.01	−0.39	0.01	−0.83	<0.01	−0.48	<0.01	−0.29	0.06	0.61	<0.01
	2%/1 mm	−0.84	<0.01	−0.36	0.02	−0.83	<0.01	−0.42	<0.01	−0.24	0.12	0.56	<0.01
V_98%_	3%/3 mm									−0.05	0.74	0.6	<0.01
	3%/2 mm									0	0.98	0.57	<0.01
	2%/2 mm									0.06	0.69	0.55	<0.01
	2%/1 mm									0.14	0.36	0.51	<0.01
V_95%_	3%/3 mm									0.06	0.72	0.6	<0.01
	3%/2 mm									0.12	0.47	0.57	<0.01
	2%/2 mm									0.17	0.28	0.54	<0.01
	2%/1 mm									0.23	0.14	0.49	<0.01

DVH, dose***–***volume histogram; PTV, planning target volume.

D_max_ is defined as the maximum point dose to the tissue, D_mean_ is the mean point dose to the tissue, V_98%_ is the volume of tissue receiving 98% of the prescription dose, and V_95%_ is the volume of tissue receiving 95% of the prescription dose.

## 4 DISCUSSION

Our results showed that the clinical impact of MLC positioning errors depends on both error type and magnitude of error. Systematic errors were more clinically significant than random errors with maximum value of similar size (“random errors of equal magnitude”) when examining changes to DVH, a phenomenon previously reported in the literature.^1^, ^2^ Although dose metrics were affected fairly linearly by both, the percentage of plans with unacceptable changes to PTV coverage remained constant for all random errors (Fig. 5).

The clinical impact of systematic errors was not only consistently greater than that of random errors of the same magnitude but also often greater than that of random errors of larger magnitude. This is consistent with the nature of the uniformly random distribution used for the current study. Because each random error added to the plan had an equal probability of falling anywhere within the minimum and maximum boundaries of the distribution, roughly 50% of all leaves would have moved less than half of the range of the distribution. Fewer leaves deviated far from their original position, thus limiting the overall effect of the errors. Had the random distribution been weighted to favor larger shifts or (equivalently) had a different distribution method been chosen, the dosimetric impact of random errors may have been greater.

Although high‐risk PTV coverage was most significantly changed by systematic errors for both V_95%_ and V_98%_, mid‐ and low‐risk PTVs were impacted as well. A decrease in mean volume was immediately observable at 3 mm systematic error and continued to trend downward as systematic errors increased. The greatest change occurred to high‐risk PTVs for V_98%_ with 10 mm systematic errors, in which the mean target volume coverage decreased 30% below the mean target volume for error‐free plans. As a comparison, mean target volume coverage for the high‐risk PTV with 10 mm random errors, evaluated at V_98%_, decreased by less than 5%. Although less pronounced, systematic errors continued to cause a greater decrease in mid‐ and low‐risk PTV coverage than did random errors.

Although systematic errors are far more problematic from a dosimetric standpoint, they are also more easily caught by portal dosimetry. Gamma analysis consistently failed in any plan containing even 3 mm of systematic error and continued to do so in plans with larger errors. Random errors are more difficult to detect within a uniform distribution of ±7 mm or less, but randomized errors also have less clinical significance within this range. As they become large enough to impact the treatment plan, detection of these errors becomes much better.

Zhen et al found that gamma passing rate, in general, correlated only weakly with clinically significant, even critical, patient errors.^17^ Their work is similar to the current study in that both examined treatment plans of patients with head and neck cancer treated with a 6‐MV beam in which errors were purposely introduced, although treatment modality differed (IMRT instead of VMAT). In addition, there were some differences in the selection of gamma criteria. We agree with their conclusion that the 3%/3 mm gamma indices in common use are not sufficiently stringent, as our analysis showed that an unacceptably large percentage of plans containing clinically significant errors passed portal dosimetry QA at these indices. For example, all plans with 7 mm random errors passed, although 45% of these contained clinically significant changes in dose to normal tissues. However, we noticed a higher correlation between the portal dosimetry pass rate and the occurrence of clinically significant errors for certain structures. Interestingly, although correlation varied across DVH metrics and the use of either random or systematic errors, the selection of gamma indices had little impact on correlation.

The recommendation of Zhen et al, as well as TG‐218, that other gamma indices be used instead of 3%/3 mm in common usage is supported by our work.^15^, ^17^, ^18^ Specifically, we found that the 3%/2 mm index recommended by TG‐218, which mostly came from experience with single‐layer MLCS, also seemed to hold for this dual‐layer system and performed well when using conventional pass/fail criteria of pixel agreement >95%. This index consistently detected clinically significant errors, including all systematic errors, while allowing an acceptable number of plans with clinically insignificant errors to pass 95% agreement criteria. If stricter detection of error plans is desired, the 2%/2 mm index failed all error plans with random errors of ≥5 mm but allowed most unmodified and 3 mm random error plans to pass (91% and 73%, respectively). The tighter index of 2%/1 mm performed poorly, failing all plans, including unmodified plans.

In comparison, the secondary portal dosimetry pass***–***fail technique continued to fail all systematic error plans. However, more random error plans were able to pass evaluation when tighter gamma indices such as 2%/1 and 2%/2 mm were used, whereas fewer passed for 3%/2 mm or 3%/3 mm indices. This occurred because at these indices the unmodified plans generally were within 97.5% or higher pixel agreement with predicted fluence, which increased the minimum necessary pixel agreement to >95.5%. Had we chosen to assume any plan with ≥95% agreement passed, regardless of the pass rate of the original plan, the pass rates for random errors at 3%/3 and 3%/2 mm would be identical to the values reported above. Finally, given that pass rate was determined solely by minimum pixel agreement, this secondary technique passed all unmodified plans at all gamma indices.

There are a few limitations associated with the current study. The research version of Eclipse for the preclinical Halcyon unit did not support delivering treatment plans with fully independent leaf motion; instead, the distal MLC layer was slaved to the proximal layer. Distal leaves were never allowed to protrude into the beam or be closer than 0.1 mm to the beam edge, which was determined solely by the edges of the proximal leaves. Therefore, errors were simulated only in the proximal layer leaf positions, and then distal leaves were adjusted to ensure that they were within the requirements imposed by Eclipse. This constraint has been removed in the clinical versions of Eclipse, which now allow fully independent motion of proximal and distal leaves.

The relatively small patient cohort and restriction to VMAT head and neck treatment plans somewhat limit the range of the study. However, we believe that the dataset of 99 unique plans largely offsets the effect of a small cohort. A previously conducted study for 168 VMAT lung treatment plans provided additional confidence that the Halcyon’s portal dosimetry is able to catch errors occurring in various treatment sites before they become clinically significant.^19^

One limitation of this study is the fact that we did not thoroughly examine the impact of MLC errors less than 3mm. This minimum was originally determined based on preliminary experiments with the Halcyon which showed that errors smaller than 3 mm did not have a noticeable impact. Because systematic error plans always failed portal dosimetry analysis, even for the smallest error considered (3 mm), additional experiments would be needed to determine the impact of errors smaller than 3 mm and if there is a range of systematic errors that do not significantly affect patient dose. We acknowledge that very large systematic errors, such as the 10 mm error used in the current study, represent a worst‐case scenario and are highly unlikely to occur in actual practice, especially considering the Machine Performance Check QA that must always be performed for the Halcyon.^20^ These large errors were included to provide contrast with 10 mm random error and to illustrate the continual deterioration of dosimetric metrics as systematic errors increase.

## 5 CONCLUSION

While MLC positioning errors in any treatment plan delivered on the Halcyon are undesirable and should be minimized when possible, the type and magnitude of errors can greatly impact the clinical significance of these errors. Systematic errors consistently introduced more clinically significant changes to dose delivered to normal tissues and to target volume coverage than did random errors of the same magnitude. Consequently, although systematic errors introduced greater dosimetric changes, they were also relatively easy to detect with portal dosimetry. Random errors were less likely to introduce clinically significant changes but also more difficult to detect.

Correct detection of clinically significant errors with portal dosimetry depends greatly on the choice of evaluation criteria. The gamma index of 3%/2 mm recommended by TG‐218 also held for this dual‐layer MLC system.

## CONFLICT OF INTEREST

This work was made possible through a partnership with Varian Medical Systems that provided access to a preclinical Halcyon. The authors have no further conflict of interest to disclose.

6
